# Severe sepsis and septic shock survival in a clinical canine model

**DOI:** 10.1186/cc13009

**Published:** 2013-11-05

**Authors:** JGMP Isola, AE Santana, GB Pereira-Neto, Rodrigo C Rabelo

**Affiliations:** 1 Universidade Estadual Paulista 'Julio de Mesquita Filho', FCAV UNESP, Jaboticabal, SP, Brazil; 2 Universidade de Brasília - FAV, Brasília, DF, Brazil; 3 Veterinary Department at AMIB (Brazilian Intensive Care Association) and Intensivet Veterinary Consulting, Brasília, DF, Brazil

## Background

Sepsis is a major cause of death in veterinary medicine, as in the human field, but there are no survival data described for this syndrome in the veterinary clinical field. This aspect challenges experimental medicine, may alter the baseline data to be applied in the human setting and could explain in part why most results obtained from laboratory research are not completely useful in the human clinical field. The purpose of this prospective observational study was to investigate the 24-hour and 30-day survival from severe sepsis and septic shock in canine septic patients that were approached with the Surviving Sepsis Campaign (SSC) bundles.

## Materials and methods

Nineteen client-owned puppies with naturally acquired parvovirus haemorrhagic gastroenteritis were classified as severe sepsis and septic shock patients and received medical care according to the guidelines proposed by the SSC. Subsequently, the 24-hour and 30-day survival was evaluated for each case. The results were statistically analysed by Fisher's exact test at a significance level of 5%.

## Results

Fifteen patients (78.9%) were admitted to the emergency department and classified as severe sepsis subjects. The mortality rate in the severe sepsis group was 33.33% (five animals), of which four animals died in the first 24 hours of admission and the other on the following day. Four dogs (21.1%) were classified as septic shock patients. The mortality rate in the septic shock group was 100%, of which two animals died in the first 24 hours of admission and two on the day after (Table 1).

**Table 1:** Severe sepsis and septic shock animals classified as nonsurvivors and survivors 24 hours and 30 days after admission

Classification	Total	Nonsurvivors 24 hours	Survivors 24 hours	*P*	Nonsurvivors 30 days	Survivors 30 days	*P*
Severe sepsis	14	4 (26.7%)	11 (73.3%)	0.557	5 (33.3%)	10 (66.7%)	0.033
Septic shock	4	2 (50.0%)	2 (50.0%)		4 (100.0%)	0 (0.0%)	
Total	19	6 (31.6%)	13 (68.4%)		9 (47.4%)	10 (52.6%)	

*P*, significance value of Fisher's exact test.

## Conclusions

The observation of clinical outcomes in this clinical canine sepsis model showed that the majority of deaths in both severe sepsis and septic shock occur within the first 24 hours. However, after 30 days there is a significant difference between both groups, showing no survival in septic shock animals. Therefore, this preliminary study suggests a new veterinary database to be applied for future human research.

